# Estimates of COVID-19 Cases and Deaths Among Nursing Home Residents Not Reported in Federal Data

**DOI:** 10.1001/jamanetworkopen.2021.22885

**Published:** 2021-09-09

**Authors:** Karen Shen, Lacey Loomer, Hannah Abrams, David C. Grabowski, Ashvin Gandhi

**Affiliations:** 1 Department of Economics, Harvard University, Cambridge, Massachusetts; 2 Department of Economics and Health Care Management, Labovitz School of Business and Economics, University of Minnesota, Duluth; 3 Department of Medicine, Massachusetts General Hospital, Boston; 4 Department of Health Care Policy, Harvard Medical School, Boston, Massachusetts; 5 Anderson School of Management, University of California, Los Angeles

## Abstract

**Importance:**

Federal data underestimate the impact of COVID-19 on US nursing homes because federal reporting guidelines did not require facilities to report case and death data until the week ending May 24, 2020.

**Objective:**

To assess the magnitude of unreported cases and deaths in the National Healthcare Safety Network (NHSN) and provide national estimates of cases and deaths adjusted for nonreporting.

**Design, Setting, and Participants:**

This is a cross-sectional study comparing COVID-19 cases and deaths reported by US nursing homes to the NHSN with those reported to state departments of health in late May 2020. The sample includes nursing homes from 20 states, with 4598 facilities in 12 states that required facilities to report cases and 7401 facilities in 19 states that required facilities to report deaths. Estimates of nonreporting were extrapolated to infer the national (15 397 facilities) unreported cases and deaths in both May and December 2020. Data were analyzed from December 2020 to May 2021.

**Exposures:**

Nursing home ownership (for-profit or not-for-profit), chain affiliation, size, Centers for Medicare & Medicaid Services star rating, and state.

**Main Outcomes and Measures:**

The main outcome was the difference between the COVID-19 cases and deaths reported by each facility to their state department of health vs those reported to the NHSN.

**Results:**

Among 15 415 US nursing homes, including 4599 with state case data and 7405 with state death data, a mean (SE) of 43.7% (1.4%) of COVID-19 cases and 40.0% (1.1%) of COVID-19 deaths prior to May 24 were not reported in the first NHSN submission in sample states, suggesting that 68 613 cases and 16 623 deaths were omitted nationwide, representing 11.6% of COVID-19 cases and 14.0% of COVID-19 deaths among nursing home residents in 2020.

**Conclusions and Relevance:**

These findings suggest that federal NHSN data understated total cases and deaths in nursing homes. Failure to account for this issue may lead to misleading conclusions about the role of different facility characteristics and state or federal policies in explaining COVID outbreaks.

## Key Points

**Question:**

How many COVID-19 cases and deaths at nursing homes were missed in the federal National Healthcare Safety Network (NHSN) reporting system owing to the delayed start in required reporting?

**Findings:**

In this cross-sectional study of 15 307 US nursing homes, approximately 44% of COVID-19 cases and 40% of COVID-19 deaths that occurred before the start of reporting were not reported in the first NHSN submission in sample states, suggesting there were more than 68 000 unreported cases and 16 000 unreported deaths nationally.

**Meaning:**

These findings suggest that federal NHSN data understate total COVID-19 cases and deaths in nursing homes and that using these data without accounting for this issue may result in misleading conclusions about the determinants of nursing home outbreaks.

## Teaser

This cross-sectional study examines the magnitude of unreported COVID-19 cases and deaths in the National Healthcare Safety Network and provides estimates of cases and deaths adjusted for nonreporting.

## Introduction

Although nursing homes have been centers for outbreaks and excess mortality from the COVID-19 pandemic, the federal government did not require nursing homes to report cases and deaths from COVID-19 until May 24, 2020, more than 3 months after the first reported nursing home outbreak at Life Care Center of Kirkland, Washington.^1,2^ In addition, in the first submission to the Centers for Disease Control and Prevention (CDC) National Healthcare Safety Network (NHSN), facilities were given the option—but were not required—to retrospectively report cases and deaths from earlier in the pandemic.^3^ For example, the Life Care Center of Kirkland reported zero cumulative COVID-19 cases in the first NHSN submission, despite a March 2020 CDC investigation identifying 81 COVID-19 cases and 23 COVID-19 deaths among residents.^4,5^ It is not known how many facilities chose to report retrospective data to the NHSN and what factors may have influenced their decisions (eg, data availability, reporting burden, reputation). As a result, although these data are widely known to undercount total cases and deaths in nursing homes, the degree of nonreporting, and thus the true impact of COVID-19 on nursing homes, remains unknown.^6,7^

In light of the federal data limitations, significant efforts have been made to provide alternative estimates of COVID-19 cases and deaths in nursing homes.^8,9^ However, these alternative estimates generally rely on a patchwork of state and local sources and have their own limitations. Data are not available for all states and include significant numbers of non–nursing home residences (eg, assisted living) in some states, and only nursing homes in others.

To our knowledge, no previous study has used the available data sources in combination with the federal data to estimate national nursing home COVID-19 cases and deaths. This study aims to fill that gap. We have 2 objectives: to compare data from state and federal sources in 20 states with state health department data to estimate the probability that a COVID-19 case or death that occurred prior to the beginning of NHSN reporting was reported to the NHSN, and to apply an extrapolation method to produce adjusted national estimates of cumulative COVID-19 cases and deaths at 2 time points, the date of the first NHSN submission (May 24, 2020), and the date of the last submission of 2020 (December 27, 2020).

## Methods

This cross-sectional study was determined not to be human participants research by the University of California, Los Angeles, institutional review board; therefore, it was exempt from further review and informed consent. This study follows the Strengthening the Reporting of Observational Studies in Epidemiology (STROBE) reporting guideline for cross-sectional studies.

### Data

This cross-sectional study used data from all US nursing homes in late May 2020. Data sources included NHSN COVID-19 Nursing Home Data set,^3^ state health department data, Center for Medicare & Medicaid Services Nursing Home Compare^10^ and Provider of Services file,^11^ Brown University’s Long Term Care: Facts on Care in the United States,^12^ and *The New York Times* COVID-19 Database.^13^

The NHSN COVID-19 Nursing Home Data set contains weekly facility-level data on new and cumulative COVID-19 cases and deaths. In the first submission on May 24, 2020, new and cumulative cases and deaths were identical, and may or may not have included retrospective cases and deaths. In all following submissions, new cases and deaths represented cases and deaths from the week ending with the submission date, while cumulative cases and deaths date back to May 24, 2020, or earlier (if the facility reported retrospectively).

To supplement the NHSN data, we collected facility-level data from 20 state health departments that required reporting of COVID-19 cases or deaths dating back to the beginning of the pandemic. We collected cumulative resident case data from 12 states and cumulative death data from 19 states, as reported between May 21 and May 29, 2020, to compare with the May 24, 2020, NHSN submissions. It is important to note that states varied substantially in the data they reported (eAppendix 1 in the Supplement). Briefly, states varied in what facility types were included (just nursing homes or other congregate care settings), what geographic information was provided, and in the completeness of their case and death data (ie, some states omitted non–laboratory-confirmed data, data from facilities below a certain case threshold, or data from transferred residents).

We constructed an algorithm to match these data using the facility name and available geographic information to national provider identifiers in the Center for Medicare & Medicaid Services Nursing Home Compare database using fuzzy-matching and geocoding techniques. This allowed us to separate nursing homes from non–nursing homes in the state health department data and also allowed us to match case and death data to facility characteristics.

We used data on overall star ratings, ownership (for-profit, nonprofit, or public), and number of beds from the March 2020 Nursing Home Compare file, chain affiliation from the 2020 Provider of Services file, and share of residents whose primary source of payment was Medicaid and share of residents who were non-White from the 2017 Long Term Care: Facts on Care in the United States dataset. Race/ethnicity was self-identified, and non-White residents were all residents who responded American Indian or Alaskan Native, Asian or Pacific Islander, non-Hispanic Black, or Hispanic. Daily data on total US population cases were obtained from *The New York Times* COVID-19 Database.

### Variables

Our main outcome was whether a resident COVID-19 case or death prior to May 24 was not reported to the NHSN in the May 24 data submission. First, we defined the adjusted total number of cases and deaths as of May 24, 2020 for each facility as the larger of what the facility reported to NHSN on May 24, 2020, and to their state health department on the nearest date for which we have data (within 5 days for all states). This measure assumes that facilities were unlikely to overreport cases or deaths and is likely conservative, since it does not include cases and deaths not reported to either source. The difference between the reported and adjusted estimates are cases and deaths that were reported to state authorities but not to the NHSN. Using this difference as the numerator, and the adjusted estimate of cases and deaths as the denominator, we calculated the percentage of cases and deaths prior to May 24, 2020, that were not reported to the NHSN.

We also examined the associations between reporting and nursing home characteristics, including ownership (for-profit, and not-for-profit), chain affiliation, size according to number of beds (<100, 100-150, 150-200, and >200), and overall star rating.

### Statistical Analysis

First, we described the composition of nursing homes overall and of facilities in the samples with state case data and with state death data. We used *t* tests of the difference in means (assuming unequal variances) for these descriptive variables between facilities included and not included in each analysis sample.

We examined variation in the percentage of cases and deaths as of May 24 that were not reported to the NHSN across facility characteristics, as well as by state. We performed linear regression of an indicator variable for nonreporting at the case or death level, where the independent variables are categorical variables for facility ownership, chain affiliation, size, star rating, and state. Then, we calculated estimated means from a model that included each of these facility characteristics separately (unadjusted sample means), as well as from a model that included all of the facility characteristics simultaneously (adjusted sample means). The overall unadjusted sample mean and SE were calculated from a model that only included a constant term.

We extrapolated our findings from the 20 sample states to the remaining states without state health department data (nonsample states) to estimate total national cases and deaths as of May 24. To do this, we used the (adjusted) linear regression estimates to estimate each nonsample state facility’s probability of nonreporting and then divided the facility’s NHSN report by this probability. Because it is not possible to estimate state fixed effects for the nonsample states, we used the case- or death-weighted mean of the sample state fixed effects from each regression (eAppendix 2 in the Supplement). The underlying assumption for the extrapolation was that facilities in sample states were equally likely to not report a case or death as facilities in nonsample states, conditional on our control variables. Insofar as this assumption was violated, it is likely that our national estimate of unreported cases is too low, because facilities in states that required early reporting would likely be most able to provide retrospective reports.

We also assessed the continued influence of unreported cases and deaths on estimates of the toll of the COVID-19 pandemic later in the year. To do this, we assumed that new cases and deaths reported to the NHSN after May 24 were accurate. To compute the count of cases and deaths at year-end, we added the NHSN estimate of cases and deaths on December 27 (the last submission of the year) to our measure of the unreported cases and deaths.

Finally, we applied an additional imputation method to obtain estimates of weekly cases and deaths prior to May 24 (rather than simply the cumulative estimate on May 24). Specifically, for each week prior to May 24, we calculate the share of pre–May 24 cases and deaths in the total population (not just nursing homes) that occurred in that week using *The New York Times* COVID-19 database. We assumed this share was the same as the share of pre–May 24 nursing home cases and deaths that occurred in that week and used these shares to distribute the pre–May 24 cases and deaths across weeks. For example, if *The New York Times* database indicated that 5% of pre–May 24 general population deaths occurred in the week ending May 10, we would assign 5% of our estimate of pre–May 24 nursing home deaths to that week. This is equivalent to assuming that the share of population cases and deaths occurring in nursing homes is constant prior to May 24.

Our primary analysis did not account for differences in state reporting requirements. To investigate how these differences might affect our estimates, we collected additional state health department data from later dates. We used these data to calculate the ratio of state estimates of post–May 24 cases and deaths to the corresponding federal estimate. If states had the exact same reporting requirements as the NHSN, we would expect these estimates to align exactly, ie, the ratio should be exactly 1. On the other hand, if state requirements were significantly more or less restrictive than the NHSN data, we would expect to see ratios significantly different from 1.

Analysis was conducted using Stata statistical software version 16.1 (StataCorp). *P* values were 2-sided, and statistical significance was set at *P* = .05. Data were analyzed from December 2020 to May 2021.

## Results

The Table provides summary statistics on the full sample of 15 415 nursing homes and 2 analysis samples: 4599 facilities in 12 states with state case data, and 7405 facilities in 19 states with state death data. We found several statistically significant differences between facilities in our analysis samples and the remaining facilities. Facilities in both analysis samples had significantly more cases and deaths (using the NHSN data) than their counterparts in nonsample states by the date of the first NHSN report (mean [SD] cases per facility, 8.1 [19.9] vs 2.4 [9.2]; *P* < .001; mean [SD] deaths per facility, 2.2 [5.9] vs 1.4 [8.1]; *P* < .001). The analysis samples also included more facilities in the Northeast and West and fewer in the Midwest, more for-profit facilities, and more facilities with 150 beds or more (Table). The star rating distributions of sample and nonsample facilities were similar.

**Table.** Means of Facility Characteristics by State Reporting Status

	All (N = 15 415)	State case data reporting			State death data reporting		
		Available (n = 4599)	Not available (n = 10 816)	*P* value for difference^a^	Available (n = 7405)	Not available (n = 8010)	*P* value for difference^a^
Cumulative COVID-19 data from NHSN, mean (SD) [IQR] per facility							
Cases							
As of May 24	4.5 (14.5) [0-0]	8.1 (19.9) [0-3]	2.4 (9.2) [0-0]	<.001	8.2 (20.3) [0-3]	2.6 (9.7) [0-0]	<.001
As of Dec 27	33.1 (31.9) [6-51]	38.7 (36.7) [8-59]	29.7 (28.1) [5-46]	<.001	39.9 (37.4) [9-61]	29.5 (28.0) [5-46]	<.001
Deaths							
As of May 24	1.6 (5.3) [0-0]	2.2 (5.9) [0-1]	1.4 (5.1) [0-0]	<.001	2.7 (7.0) [0-1]	0.6 (2.5) [0-0]	<.001
As of Dec 27	6.6 (9.0) [0-10]	7.6 (10.1) [0-11]	6.2 (8.5) [0-9]	<.001	7.8 (10.5) [1-11]	5.4 (7.1) [0-8]	<.001
Facility characteristics, No. (%)^b^							
Region							
Northeast	2542 (16.5)	1135 (24.7)	1407 (13.0)	<.001	2414 (32.6)	128 (1.6)	<.001
Midwest	5033 (32.7)	438 (9.5)	4595 (42.5)	<.001	718 (9.7)	4351 (53.9)	<.001
South	5446 (35.3)	1542 (33.5)	3904 (36.1)	.002	2789 (37.6)	2657 (33.2)	<.001
West	2387 (15.5)	1484 (32.3)	903 (8.4)	<.001	1484 (20.0)	903 (11.3)	<.001
Ownership or chain affiliation							
For-profit	10 743 (69.9)	3407 (74.1)	7336 (67.9)	<.001	5425 (73.3)	5318 (66.4)	<.001
Chain	8857 (59.4)	2690 (59.8)	6167 (59.3)	.55	4056 (56.1)	4801 (62.6)	<.001
Beds, No.							
<100	7690 (50.0)	2176 (47.3)	5514 (51.0)	<.001	3023 (40.9)	4667 (58.3)	<.001
100-150	5120 (33.3)	1556 (33.8)	3564 (33.0)	.31	2630 (35.6)	2490 (31.1)	<.001
150-200	1633 (10.6)	572 (12.4)	1061 (9.8)	<.001	1012 (13.7)	621 (7.8)	<.001
≥200	972 (6.0)	295 (6.4)	677 (6.3)	.46	740 (10.0)	232 (2.9)	<.001
Star rating							
1	2657 (17.5)	771 (17.0)	1886 (17.8)	.29	1232 (16.9)	1425 (18.1)	.05
2	2974 (19.6)	889 (19.6)	2085 (19.3)	.97	1433 (19.6)	1541 (19.6)	.91
3	2759 (18.2)	832 (18.3)	1927 (18.2)	.71	1329 (18.2)	1430 (18.2)	.93
4	3244 (21.5)	926 (20.4)	2318 (21.8)	.07	1540 (21.1)	1704 (21.7)	.43
5	3520 (23.2)	1119 (24.7)	2401 (22.6)	.004	1762 (24.2)	1758 (22.4)	.008

Abbreviations: IQR, interquartile range; NHSN, National Healthcare Safety Network.

^a^ *P* values are results from a 2-sample *t* test for a difference in means with unequal variances between included and excluded facilities.

^b^ Means of categorical variables for region, ownership, chain affiliation, number of beds, and star rating are expressed as percentages.

**Figure 1.**
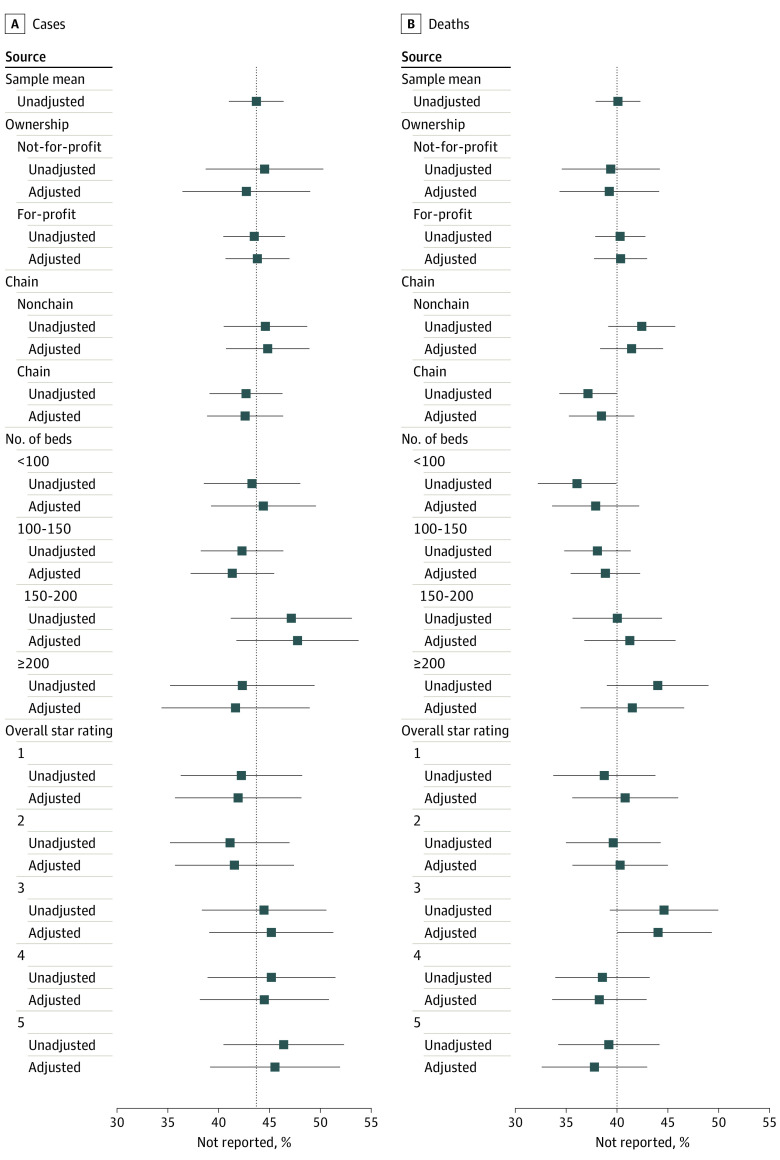
Percentages of Cases and Deaths as of May 24 Not Reported to the National Healthcare Safety Network by Facility Characteristic Estimated means (in percentages) from case- and death-level linear regression of the probability of not being reported to National Healthcare Safety Network on facility characteristics, in which SEs are clustered by facility. Squares indicate estimates; whiskers, 95% CIs.

As presented in Figure 1, a mean (SE) of 43.7% (1.4%) of cases and 40.0% (1.1%) of deaths that occurred prior to May 24 were not reported to the NHSN in the analysis samples. Figure 1 also presents unadjusted and adjusted means from a linear regression of the share of cases and deaths that were not reported on facility ownership type, chain affiliation, size, and overall star rating. The adjusted means for the included covariates were between 40% and 50% for cases and between 35% and 45% for deaths. We found no statistically significant differences along these characteristics.

**Figure 2.**
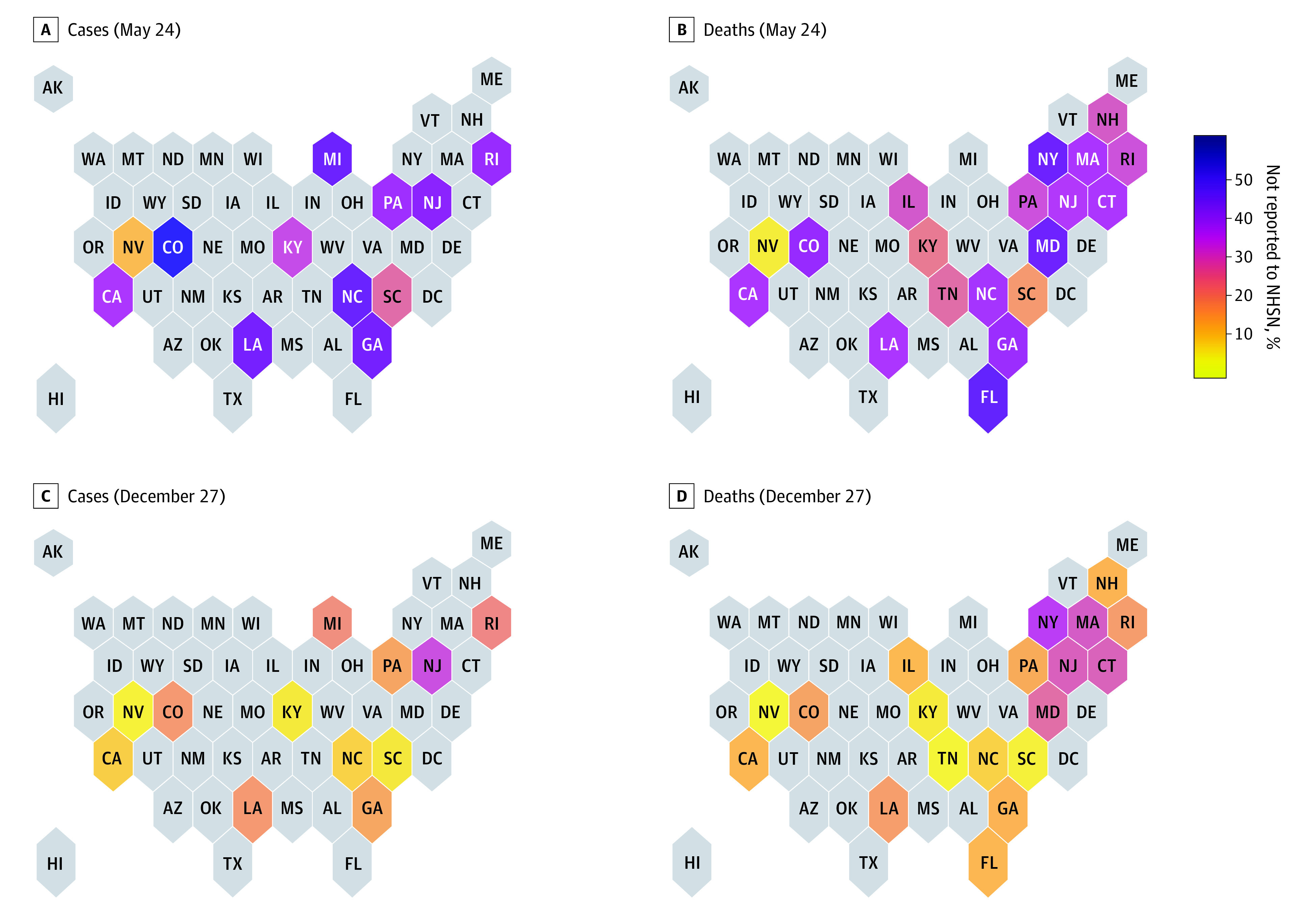
Percentage of Cases and Deaths Not Reported to the National Healthcare Safety Network (NHSN) in Sample States by State Each state is shaded according to the share of deaths as of May 24 (A and B) or December 27 (C and D) that were not reported to the National Healthcare Safety Network.

Figure 2, A and B, summarize the percentage of cases and deaths that were unreported as of May 24 by state. We found more variation by state than by facility characteristic: in most of our sample states, between 40% and 60% of cases as of May 24 were unreported, and between 30% and 50% of deaths as of May 24 were unreported. However, some of this variation may be attributable to differences in state reporting requirements. Importantly for our extrapolation assumption, we did not find much systematic regional correlation in this measure as of May 24.

Figure 2, C and D, show the impact of these unreported cases and deaths with year-end totals using data from December 27 (the last NHSN submission in 2020). The percentages of cases and deaths that were unreported were reduced by year-end (the overall mean in sample states was 13.9% of cases and 18.7% of deaths), reflecting the continued toll of the pandemic on nursing homes after the beginning of reliable reporting. There was also clear regional correlation in these year-end percentages, with states in the Northeast having the highest percentages, meaning that the delay in required reporting had the greatest impact on year-end totals in these states.

Using the raw NHSN data would imply that similar numbers of nursing home residents died in New York and California in 2020 (5776 in New York and 5633 in California, equating to 5.0 deaths per 100 beds in New York and 4.8 deaths per 100 beds in California). However, after accounting for unreported deaths, we estimate that nursing homes in New York experienced 9276 deaths (8.1 deaths per 100 beds), compared with 6487 in California (5.5 deaths per 100 beds). In addition to the aggregate estimates, our facility-level corrections are available online.^14^

**Figure 3.**
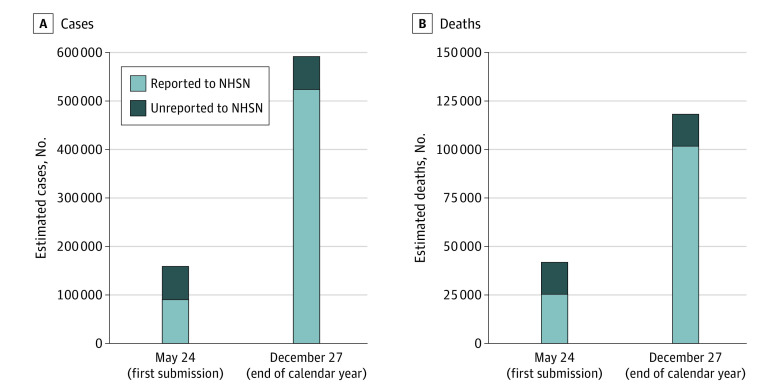
National Estimates of Cumulative Nursing Home Cases and Deaths This figure reports estimates of cumulative national cases and deaths in nursing home settings on the date of the first National Healthcare Safety Network (NHSN) submission (May 24) and the end of 2020 (December 27). Raw NHSN estimates are shown in light blue, and estimates of the cases and deaths unreported owing to the delay in required reporting (derived from state health department data from a sample of 20 states and extrapolated to all states) are shown in dark blue.

Figure 3 shows the result of extrapolating the probability of nonreporting of pre–May 24 cases and deaths to nonsample states to produce national estimates of unreported cases and deaths. There were 90 264 cases and 25 355 deaths reported nationwide in the first NHSN submission on May 24. By using our adjusted regression to estimate the share of cases and deaths that were not reported at each nonsample state facility, we estimate that 68 613 cases and 16 623 deaths were omitted in the first NHSN submission owing to the lack of required retrospective reporting, implying that a mean of 43.2% of cases and 39.6% of deaths were omitted nationally. By adding these undercount estimates to the December 27 totals (the last NHSN submission of 2020), we estimate that the year-end total nursing home case count was 592 629, and the death count was 118 335. Unreported cases and deaths accounted for 11.6% and 14.0% of these totals, respectively.

**Figure 4.**
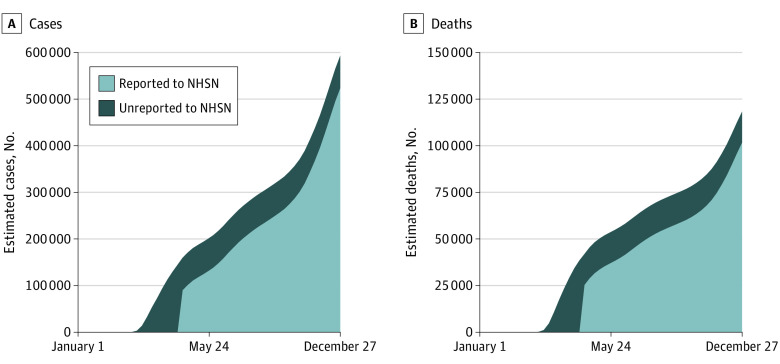
Estimated and Reported National Cumulative Cases and Deaths in Nursing Homes The dark blue area shows cumulative cases and deaths using our adjusted measure, which uses the share of nonreporting in sample states to compute an estimate of cumulative cases and deaths before May 24 then uses data from *The New York Times*^13^ on population cases and deaths to distribute this estimate across weeks prior to May 24. The light blue area shows cumulative cases and deaths using the National Healthcare Safety Network data.

Finally, Figure 4 shows these estimates in the context of the evolution of the pandemic by imputing the time pattern of cases and deaths before May 24 using case and death data for the general population. The delay in required reporting means that the NHSN data miss a significant period of the pandemic, in which cases and deaths were increasing more rapidly than any other point in 2020 except during the wave in the final months of the year.

eAppendix 3 in the Supplement shows the result of comparing state and federal data collected later in the pandemic. Ratios that are greater than 1 indicate that state data report higher cases and deaths compared with federal data, whereas ratios less than 1 indicate state data report lower cases and deaths compared with federal data. We found that for several states (ie, California, Colorado, Georgia, Kentucky, and Pennsylvania), the state and federal data for cases and deaths were in agreement after May 24, with ratios between 0.88 and 1.09. In other states, the state data had higher reported cases and deaths than the federal data (ie, Connecticut, Florida, Massachusetts, New Jersey, and Rhode Island), with ratios ranging from 1.20 to 1.57, and, in a few states, the state data are lower than the federal data (ie, New Hampshire, Tennessee, New York), with ratios ranging from 0.61 to 0.82.

## Discussion

This cross-sectional study used data from 20 state health departments to evaluate and supplement federal data on COVID-19 cases and deaths in nursing homes. We estimate that 44.7% of COVID-19 cases and 40.0% of COVID-19 deaths occurring prior to May 24 were not reported in the first NHSN submission. These unreported cases and deaths had a significant influence on our estimates of total cases and deaths attributable to COVID-19 in nursing homes, accounting for 11.6% of cases and 14.0% of deaths in the year-end totals.

We did not find differences in nonreporting by facility characteristics (ie, region, ownership, chain affiliation, or star rating) as of May 24. This implies that facilities of all types omitted previous cases and deaths in the first NHSN submission. This may demonstrate a widespread inability of nursing homes to reliably collect data early in the pandemic or that pressures to report fewer cases and deaths were common to all facilities.

Accounting for this delay is important when comparing the toll of the pandemic across places. Consistent with the fact that states in the Northeast were hit hardest in the early months of the pandemic but generally experienced lower case and death rates in later months, we found that unreported cases and deaths represented a significantly larger share of year-end totals in the Northeast than in the South and West, where most cases and deaths occurred later.

### Limitations

This study has some limitations. Some limitations of our estimates are the use of extrapolation from sample states to nonsample states, potentially differing reporting requirements across states, and the fact that our analysis does not include cases and deaths that were not reported to state or federal authorities. We also did not analyze reporting of staff cases and deaths. Regarding extrapolation, although facilities in sample states and nonsample states differed significantly on several important characteristics (eg, region, ownership, size), we do not find that these characteristics were associated with the likelihood of nonreporting; thus, we believe our extrapolation is reasonable. Regarding state reporting requirements, the fact that our estimates were similar for both cases and deaths is reassuring. We also used later state reports to assess the degree to which these differences may have affected our estimates. We found that some states may have defined cases and deaths more broadly than the NHSN, and others may have used more conservative definitions. For example, New York’s health department excluded resident deaths that took place outside of the facility, such as when a patient died after being discharged to a hospital.^15^ These findings have implications for the interpretation of our estimates: in states with broader reporting requirements, our undercount estimate may be overstated, while in states with more restrictive definitions, our undercount estimate may be understated.

## Conclusions

The findings of this cross-sectional study suggest that federal NHSN data understated total COVID-19 cases and deaths in nursing homes. To date, both academic and policymakers’ analyses of facility-level determinants of infections and mortality have likely been limited owing to the reliance on federal estimates.^16,17,18^ In particular, use of the unadjusted federal data may help explain why some reports found an association between lower-rated nursing homes and COVID-19 outbreaks (a conclusion that guided early enforcement actions against nursing homes), while others did not.^19,20,21,22^ Our data, which we have made publicly available,^14^ also offer the ability to credibly study the associations of facility responses and state and federal policy in the early months of the pandemic with slowing the spread in nursing homes, which is not possible with the federal data owing to missing data.

## Notes

Supplement. **eAppendix 1.** Further Information on State Health Department Data **eAppendix 2.** Further Information on the Extrapolation Method **eAppendix 3.** Further Information on Differences in State Reporting in State Data Using More Recent State Reports
