# Supplementary material for: Estimates of COVID-19 Cases and Deaths Among Nursing Home Residents Not Reported in Federal Data
Source: JAMA Netw Open. 2021 Sep 9;4(9):e2122885. doi: 10.1001/jamanetworkopen.2021.22885 (PMC8430452; doi:10.1001/jamanetworkopen.2021.22885)
Supplement: Supplement. — eAppendix 1. Further Information on State Health Department Data eAppendix 2. Further Information on the Extrapolation Method eAppendix 3. Further Information on Differences in State Reporting in State Data Using More Recent State Reports [file jamanetwopen-e2122885-s001.pdf]

# Supplemental Online Content

Shen K, Loomer L, Abrams H, Grabowski DC, Gandhi A. Estimates of COVID-19 cases and deaths among nursing home residents not reported in federal data. *JAMA Netw Open*. 2021;4(9):e2122885. doi:10.1001/jamanetworkopen.2021.22885

**eAppendix 1.** Further Information on State Health Department Data
**eAppendix 2.** Further Information on the Extrapolation Method
**eAppendix 3.** Further Information on Differences in State Reporting in State Data Using More Recent State Reports

This supplemental material has been provided by the authors to give readers additional information about their work.

**eAppendix 1.** Further Information on State Health Department Data

The below table summarizes the facility-level data we collected from state health departments. The second column notes the date of the state report that we used: all were within 1 week of May 24, 2020. Because some state data includes data for other types of long-term care facilities or congregate living settings (e.g. assisted living facilities), our first step in cleaning the data was to match each facility in the state data to the CMS Nursing Home Compare database in order to exclude non-nursing homes from our estimates. We performed this matching using an algorithm that gave each potential match between  a score based on returning the same establishment using the Google Maps API, a fuzzy-matching score of the facility names, and whether the two facilities shared the same geographic identifiers, where available (address, city, or county). We hand-checked all matches below a certain score threshold. The third column of Table A1 documents which variables were available in the state data to match, and whether the state data included non-nursing homes. In some cases, the state data included non-nursing homes, but also included a facility type variable, allowing us to simply restrict the data to nursing homes. In other states, non-nursing homes were included and there was no facility type variable. For these states, where possible, we gathered data on the names and locations of licensed assisted living facilities to improve the matching process (we then performed the matching algorithm on the set of nursing homes from Nursing Home Compare and the other facilities from the state data).

Finally, the fourth column notes details about the case and death measures that states reported. We attempt to use the closest measure to total confirmed and probable cases and deaths among residents that is available; however, there are some notable differences across states in what this measure is. For example, states report only laboratory-confirmed cases and deaths, others only report cases and deaths at facilities with “outbreaks” (usually defined as a certain number of cases in a given set of days), and some states may be missing cases and deaths for transferred residents or that occurred before a certain date. The next section discusses implications of these reporting differences for our results. Some states censor their data or provide the data in ranges: in these cases, we use the midpoint of the range. We do not use measures of cases from IL and TN because they include both residents and staff and are thus likely to significantly overstate cases but not deaths (since deaths among residents is overwhelmingly higher than deaths among staff), and we do not use cases from MA because they report cases in very coarse buckets. Two states (MD and TN) remove facilities from the data if a certain number of days has passed since the facility last reported a case. For these states, we pull the entire history of reports, and use the last observation of each facility.

**Table A1: Data and matching details for state health department data**

| State | Date Used | Matching details | Data details (measures, censoring) |
|---|---|---|---|
| CA | 5/24/2020 | Facility name and county. SNFs only. | Cumulative confirmed resident cases; cumulative resident deaths. Includes deaths outside the facility if the death occurred within the 14-day bed hold period after the resident transferred from the SNF. Numbers less than 11 are censored. |
| CO | 5/27/2020 | Facility name and county. Data includes other settings, but has facility type variable. | Cumulative confirmed and probable resident cases; cumulative resident deaths for facilities with outbreaks (two or more confirmed cases in residents with onset in a 14 day period). |
| CT | 5/27/2020 | Facility name. Nursing homes only. | Cumulative confirmed resident cases; cumulative confirmed and probable resident deaths. |
| FL | 5/22/2020 | Facility name and county. Data includes other LTCs and does not have facility type variable; to improve matching, we collected data on universe of ALFs in FL. | Cumulative resident deaths. |
| GA | 5/22/2020 | Facility name, address, city, county. Data includes other facility types, but facility type is noted. | Cumulative resident positive cases; cumulative resident deaths (including deaths at locations other than the facility). |

© 2021 Shen K et al. *JAMA Network Open.*

| | | | |
|---|---|---|---|
| IL | 5/23/2020 | Facility name and county. Data includes other LTCs and does not have facility type variable; to improve matching, we collected data on universe of ALFs in IL. | Cumulative deaths (may include staff members; we do not use cases because staff members may be included). |
| KY | 5/22/2020 | Facility name and county. Data includes other LTCs and does not have facility type variable; to improve matching, we collected data on universe of ALFs in KY. | Cumulative positive residents; cumulative resident deaths (beginning March 7, 2020). |
| LA | 5/25/2020 | Facility name and parish. Nursing homes only. | Cumulative resident cases; cumulative resident deaths. |
| MA | 5/27/2020 | Facility name and county. Nursing homes and rest homes only. | Cumulative resident deaths. Facilities with 2+ cases only. Numbers under 5 are censored. (We did not use case data because numbers under 30 are censored). |
| MD | 5/27/2020* | Facility name only. Data includes all congregate living facilities; to improve matching, we collected data on universe of ALFs in MD. | Cumulative confirmed resident cases and deaths (beginning April 15, 2020). *Used weekly reports from April 29-May 27, because facilities are removed after 28 days. |
| MI | 5/28/2020 | Facility name and county. SNFs only. | Cumulative confirmed resident cases. |
| NC | 5/22/2020 | Facility name and county. All Data includes other congregate living settings, but has facility type variable. | Cumulative resident cases and deaths. Facilities with outbreaks only (two or more laboratory-confirmed cases). |
| NH | 5/29/2020 | Facility name only. Data includes other congregate living living settings, but has facility type variable. | Cumulative resident cases; cumulative deaths. |
| NJ | 5/22/2020 | Facility name and county. Data includes other LTCs;  to improve matching, we supplemented the NH universe data with data on MD ALFs. | Cumulative confirmed resident cases; cumulative confirmed resident deaths.. |
| NV | 5/22/2020 | Facility name and county. Data includes other settings, but has facility type variable. | Cumulative confirmed resident cases; cumulative confirmed resident deaths. |
| NY | 5/21/2020 | Facility name and county. Data includes ALFs, but in separate table. | Cumulative confirmed and presumed resident deaths. Only includes deaths occurring at the facility. |
| PA | 5/22/2020 | Facility name, city, county. Data includes ALFs, but in separate table. | Cumulative resident cases; cumulative deaths. Numbers less than 5 are censored. |
| RI | 5/22/2020 | Facility name and city. Data includes ALFs, but in separate table. | Cumulative resident cases; cumulative resident deaths. Facilities with 2 or more resident cases only. |
| SC | 5/22/2020 | Facility name, address, city. Facility type is noted. Data includes other LTCs, but facility type variable. | Cumulative confirmed resident cases; cumulative confirmed resident deaths. |
| TN | 5/22/2020* | Facility name, city county. Other facility types included. | Cumulative deaths (includes both residents and staff, so we do not use cases). Facilities with 2 or more confirmed cases among residents and staff within 28 days. *Used weekly reports from April 18-May 22, because facilities are removed after 28 days. |

© 2021 Shen K et al. *JAMA Network Open.*

## **eAppendix 2.** Further Information on the Extrapolation Method

Our extrapolation method relies on the assumption that the degree of under-reporting in non-sample states was similar to the degree of under-reporting in sample states, conditional on observable characteristics. To extrapolate data from our sample states to the states where we do not have state department data as of May 24, we estimate a predicted probability of non-reporting for cases and for deaths for each facility, and divide the facility's reported estimate of pre-May 24 cases or deaths by this predicted probability.

The figure below shows our estimates for sample states, prior to any extrapolation.

**Figure A1: Estimated cumulative cases and deaths in all sample states on the date of the first NHSN submission (May 24) and the end of 2020 (Dec 27)**

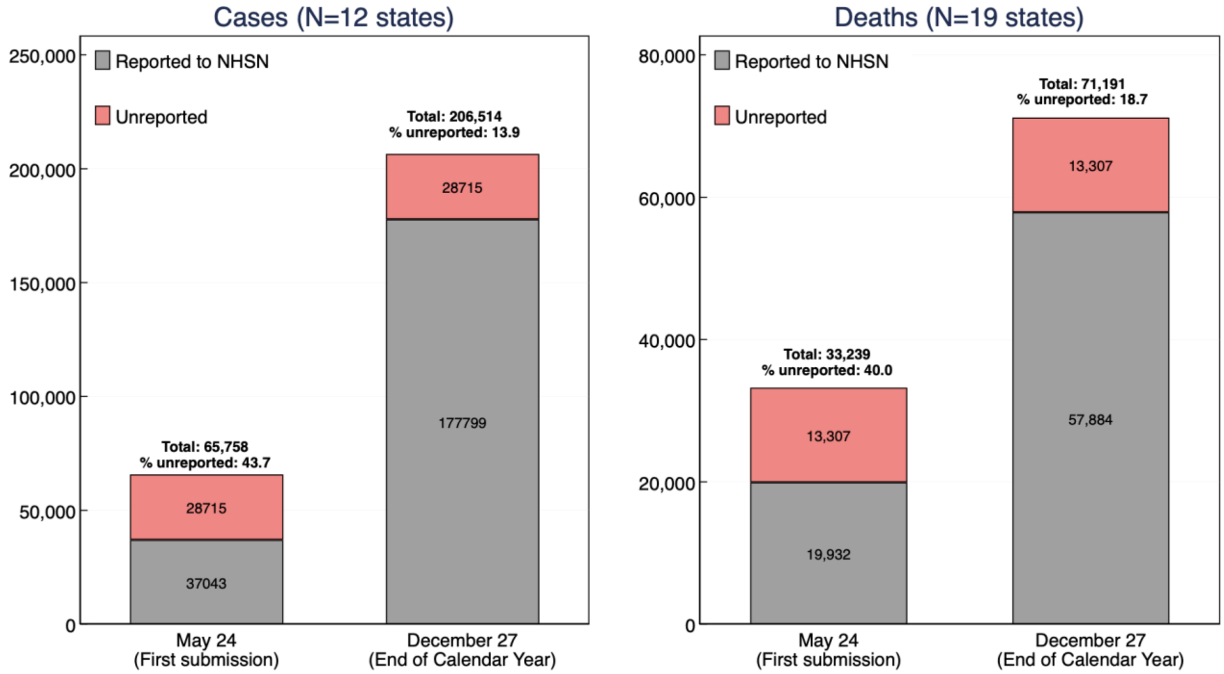


Our extrapolation method would be valid under a model of under-reporting where facilities in sample states and non-sample states were equally likely to omit retrospective cases and deaths in their first NHSN submissions. In unreported results, we assessed the reasonability of this assumption, by calculating the under-reporting percentages in each sample state separately. We found that most states appear to fall in a range of 60-100% for both cases and deaths. In addition, Figure 2 in the main text shows that there does not seem to be much systematic variation by other characteristics.

© 2021 Shen K et al. *JAMA Network Open.*

**eAppendix 3.** Further Information on Differences in State Reporting in State Data Using More Recent State Reports

An important caveat to our results is different state health departments may have differed in what cases and deaths they reported. For example, NY has been criticized for not including resident deaths that occurred outside the facility. Some other states only report data for facilities with a certain number of cases in a given timeframe. In both of these cases, the May 24 state data would thus still understate the true number of nursing home cases and deaths in these states. In this section, we collected later state reports to understand the impact of state reporting differences on our results. If the state and federal reporting requirements were the same, the counts of new cases and deaths after May 24 should match in the state and federal data. Table A2 compares state and federal counts of cases and deaths after May 24 using later state reports. We find that for several states (CA, CO, GA, KY, PA), the state and federal data for cases and deaths align quite well after May 24. In some other states, the state data appears to outpace federal data (CT, FL, MA, NJ, RI), suggesting that the undercount in these states may be somewhat overstated. Finally, in a few states, the state data is notably lower than the federal data (NH, TN, NY). This suggests that the actual undercount in these states are likely higher than implied by Figure 2, implying that the true toll in New York was likely even higher than what is reported in Figure 2. We note that using this table to understand the true size of the undercount assumes that reporting differences have a constant effect over time, which may not be true depending on the nature of the reporting difference. For example, Kentucky may be missing cases and deaths in their early data because they started reporting on March 7, but their more recent data may be unbiased.

**Table A2: Ratio of state to federal data on cases and deaths after May 24 using new state reports**

| State | Date used | State / federal Deaths | State / federal Cases |
|---|---|---|---|
| CA | 11/29/20 | 1.00 | 0.98 |
| CO | 11/22/20 | 0.90 | 1.03 |
| CT | 11/29/20 | 1.20 | |
| FL | 11/29/20 | 1.36 | |
| GA | 11/29/20 | 1.02 | 1.13 |
| IL | 11/29/20 | 0.97 | 1.45 |
| KY | 11/15/20 | 0.88 | 1.09 |
| LA | 11/22/20 | 0.82 | 1.06 |
| MA | 11/29/20 | 1.33 | |
| MD | 11/22/20 | 0.95 | |
| MI | 11/29/20 | | 1.32 |
| NC | 12/6/20 | 0.81 | 0.84 |
| NH | 11/15/20 | 0.66 | 0.61 |
| NJ | 7/12/20 | 1.22 | 1.47 |
| NV | 12/6/20 | 1.27 | 0.82 |
| NY | 11/29/20 | 0.82 | |
| PA | 11/29/20 | 0.98 | 0.89 |
| RI | 11/22/20 | 1.57 | 1.18 |
| SC | 11/29/20 | 1.04 | 0.80 |
| TN | 12/20/20 | 0.61 | 0.72 |
| Overall | | 1.08 | 1.09 |

© 2021 Shen K et al. *JAMA Network Open.*
